# Dominoes with interlocking consequences triggered by zinc: involvement of microelement-stimulated MSC-derived exosomes in senile osteogenesis and osteoclast dialogue

**DOI:** 10.1186/s12951-023-02085-w

**Published:** 2023-09-23

**Authors:** Shi Yin, Sihan Lin, Jingyi Xu, Guangzheng Yang, Hongyan Chen, Xinquan Jiang

**Affiliations:** 1 Department of Prosthodontics, Shanghai Ninth People’s Hospital, Shanghai JiaoTong University School of Medicine, No. 639 Zhizaoju Road, Shanghai, 200011 People’s Republic of China; 2 College of Stomatology, Shanghai JiaoTong University, No. 639 Zhizaoju Road, Shanghai, 200011 People’s Republic of China; 3 National Center for Stomatology, National Clinical Research Center for Oral Diseases, No. 639 Zhizaoju Road, Shanghai, 200011 People’s Republic of China; 4 Shanghai Engineering Research Center of Advanced Dental Technology and Materials, Shanghai Key Laboratory of Stomatology, Shanghai Research Institute of Stomatology, No. 639 Zhizaoju Road, Shanghai, 200011 China

**Keywords:** Senile osteogenesis, Exosomes, Cellular communication, Orthopedic implant, Zinc

## Abstract

As societal aging intensifies, senile osteoporosis has become a global public health concern. Bone microdamage is mainly caused by processes such as enhancing osteoclast activity or reducing bone formation by osteoblast-lineage cells. Compared with young individuals, extracellular vesicles derived from senescent bone marrow mesenchymal stem cells(BMSCs) increase the transient differentiation of bone marrow monocytes (BMMs) to osteoclasts, ultimately leading to osteoporosis and metal implant failure. To address this daunting problem, an exosome-targeted orthopedic implant composed of a nutrient coating was developed. A high-zinc atmosphere used as a local microenvironmental cue not only could inhibit the bone resorption by inhibiting osteoclasts but also could induce the reprogramming of senile osteogenesis and osteoclast dialogue by exosome modification. Bidirectional regulation of intercellular communication via cargoes, including microRNAs carried by exosomes, was detected. Loss- and gain-of-function experiments demonstrated that the key regulator miR-146b-5p regulates the protein kinase B/mammalian target of rapamycin pathway by targeting the catalytic subunit gene of PI3K–PIK3CB. In vivo evaluation using a naturally-aged osteoporotic rat femoral defect model further confirmed that a nutrient coating substantially augments cancellous bone remodeling and osseointegration by regulating local BMMs differentiation. Altogether, this study not only reveals the close link between senescent stem cell communication and age-related osteoporosis but also provides a novel orthopedic implant for elderly patients with exosome modulation capability.

## Introduction

Nowadays, life expectancy has been prolonged, but the corresponding quality of life brought by longevity itself has not been satisfied [1, 2]. Senile osteoporosis has become a major health problem worldwide. It is a highly age-related disease that occurs in most people over the age of 65, and mainly manifests as the attenuation of cortical and trabecular bone [3, 4]. Metal implants typically fail to appropriately address this issue, resulting in minimal opportunities for treatment, including perforated metal implant prostheses, bone-anchored hearing aids, and dental implants [5].

Human aging is a progressive degenerative state accompanied by the depletion of tissue stem cells, matrix changes, and changes in intercellular communication [1, 6]. The imbalanced differentiation of bone marrow mesenchymal stem cells (BMSCs) into osteoclasts at the expense of osteoblasts has important implications for age-related bone marrow obesity and osteoporosis [7, 8]. If this orchestrated response becomes dysregulated, a transient increase in osteoclast precursors and dysfunction of osteogenesis occur [9, 10]. Therefore, concomitantly controlling the BMSC-bone marrow monocytes (BMMs) balance and promoting regeneration is necessary in senile osteoporosis.

Aging-associated secretory phenotype (SASP) secretion by BMSCs increases with age and can release numerous bioactive substances that alter the functions of stem and progenitor cells [11]. Among them, exosomes are extracellular vesicles (EVs) of 30–150 nm in size that are released from cells and are considered ubiquitous mediators of cell-to-cell interactions and communication [12–14].They can also be considered part of SASP, which can play a role in premature senescence induced by senescent BMSCs [2, 15]. In addition, SASP amplifies senescence signals in an autocrine, paracrine, and endocrine manner with the participation of EVs [16].

Numerous functional molecules, including proteins, microRNAs (miRNAs), and DNA, are considered the main functional cargo of exosomes, and BMSCs can change the biological behavior of recipient cells by transmitting molecular information [17, 18]. The cell contents also undergo dramatic changes with age. Hamrick showed that muscle-derived EVs carrying senescence-related miRNAs can induce cellular senescence in BMSCs [19], revealing a potential pattern of organ-to-organ crosstalk. Among them, candidate miRNAs that mediate the transient activation of osteoclasts induced by aged BMSC-exosomes can be screened based on miRNA expression profiling [20]. Therefore, we hypothesized that altering the miRNA content released by aged BMSC-EVs could impact the BMSC-BMM tandem mechanism in the bone marrow, alleviating the osteogenesis-osteoclast imbalance and thereby inducing osseointegration in aging rats with osteoporosis.

Studies have found that adequate intake of macronutrients and trace elements, including vitamins D and K and nutrients such as calcium, zinc, and copper, is an important factor in bone development and regeneration [21, 22]. Among them, zinc is a cofactor of metalloenzymes that grow along the long axis of the bone. Zinc deficiency affects the integrity of bone tissue, reduces the synthesis of collagenase, and affects alkaline phosphatase activity [23]. With increasing age, the intake of nutrients decreases and absorption slows [24]. Zinc has been confirmed to have a strong correlation with age-related changes in bone mass, and the number of patients with zinc deficiency is increasing daily [21, 23]. Therefore, developing new strategies to protect zinc against clearance in senile osteoporosis is a key technology to overcome these challenges. In this study, a zinc-modified metal implant was created using micro-arc oxidation to improve its sustained release, and a natural aging rat model was established to test the efficacy of the implant for treatment of osteoporosis. This study aimed to further verify whether the improvement of trace element content in situ could affect cellular communication through exosomes through the implantation of knee joints in elderly rats, thus improving the success rate of metal implant therapy in elderly people.

**Fig. 1:**
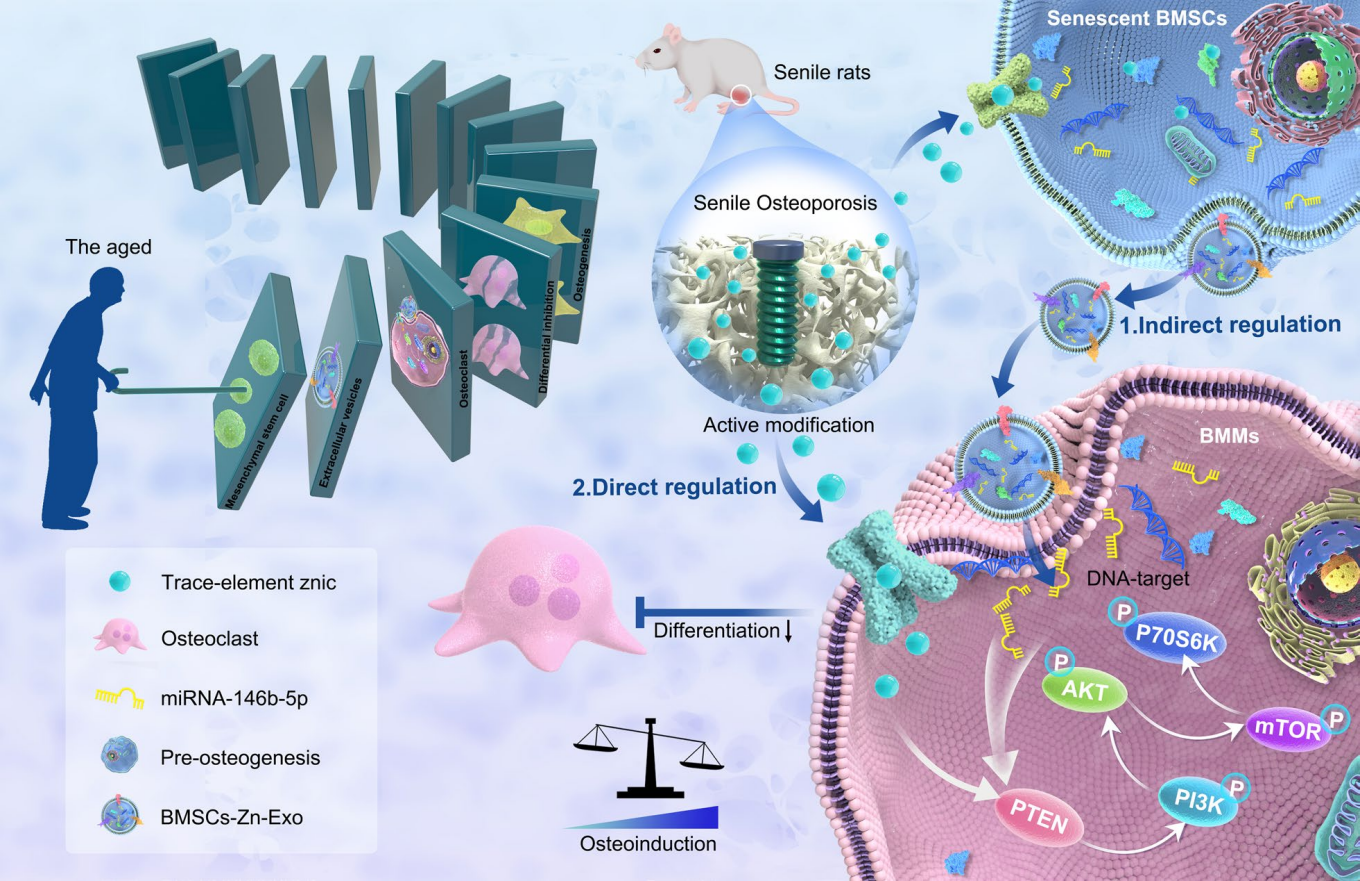
Like a domino, senile osteoporosis patients increase the expression of trace elements by engineering the microenvironment in which the mesenchymal stem cells are located. The altered exosome content secreted by engineered aging BMSCs directly affects the downstream BMSC-BMMs intercellular communication, and the transient increase in osteoclasts is abolished. Finally, the local osteogenic differentiation ability of senile osteoporosis was improved, and miRNA146b-5p was verified as a key regulator, its downstream signaling AKT/M-Tor pathway was detected

In this study, exosomes that mediate cell communication in senescent BMSCs were extracted, and the in vitro effects of aged exosomes (A-EVs) and aged exosomes with zinc (AZ-EVs) on BMM osteoclast differentiation under a high-zinc atmosphere were compared. The candidate miRNA146b-5p that may mediate osteoclast differentiation was screened based on the miRNA expression profile, and its downstream signaling pathway was detected, confirming that it inhibits the excessive differentiation of BMMs into osteoclasts by activating the protein kinase B (AKT)-mammalian target of rapamycin (mTOR) signaling pathway. In vivo, we tested whether a high zinc environment could indeed exacerbate osseointegration in aging osteoporosis (Fig. 1).

## Results and discussion

### Anti-osteoclastogenesis properties of nutritional zinc in vitro

**Fig. 2:**
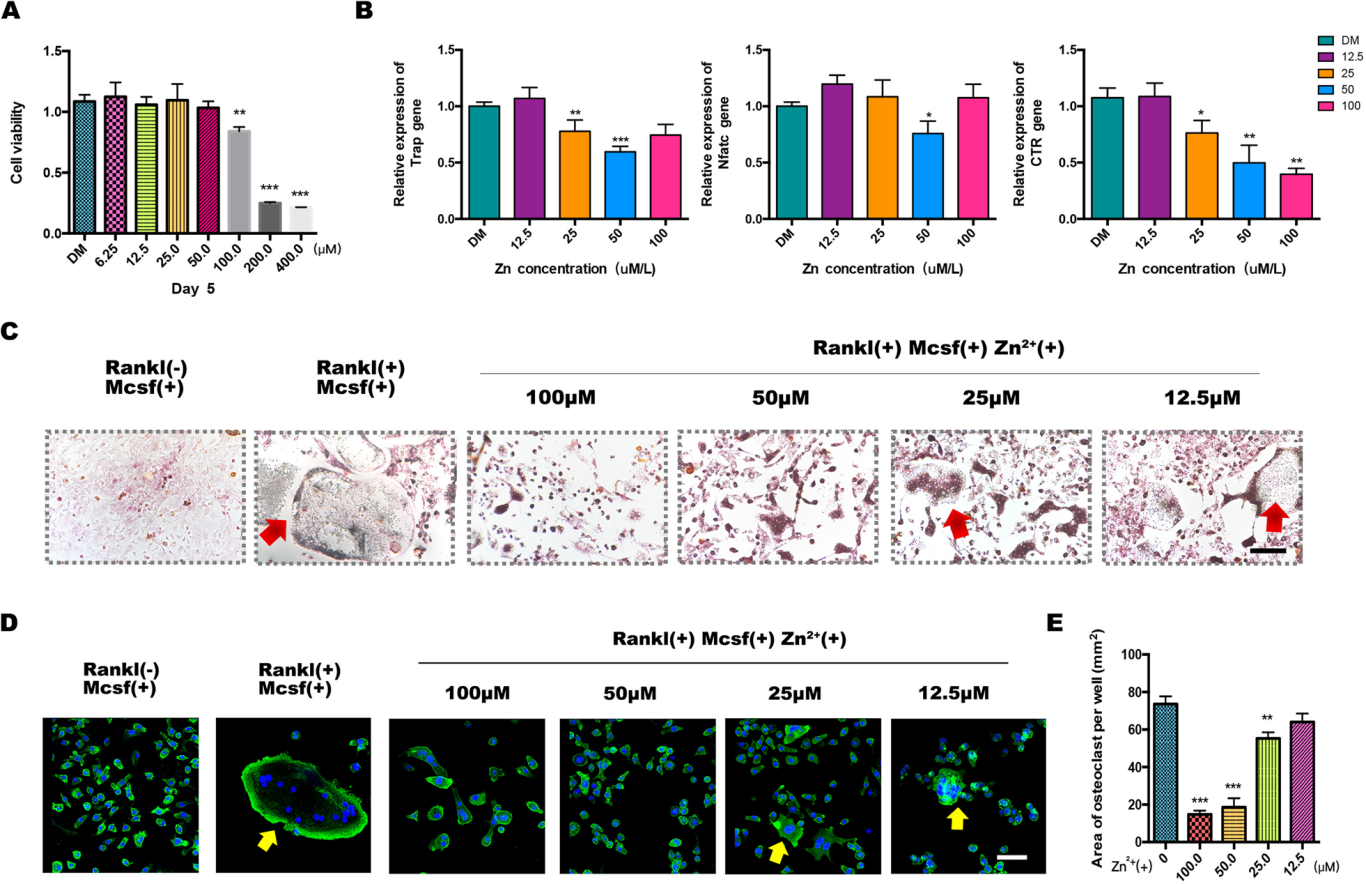
In vitro evaluation of the effects of zinc (Zn^2+^)-mediated osteoclastogenesis. **a** Cell counting kit-8 assay of the survival of bone marrow monocytes (BMMs). **b** qPCR analysis of osteoclast gene expression in BMMs. **c** Trap staining of osteoclasts induced by different doses in a Zn^2+^-enriched environment. **d**, **e** Immunofluorescence staining of actin ring formation and statistical analysis (n = 3, *p < 0.05, **p < 0.01)

Although calcium is the primary microelement for preventing bone loss, zinc, one of the most important microelements, may also be involved in the development of degenerative bone diseases. To clarify the role of zinc in osteoclastic differentiation, a cell counting kit-8 (CCK-8) assay was conducted to determine cell proliferation on day 5. The results showed that before induction of BMMs differentiation into osteoclasts, the addition of an appropriate amount of zinc trace elements had no significant effect on the basic life activities of the cells. When the zinc ion concentration reached 100 μM, the cell proliferation was significantly inhibited. When the zinc ion concentration reached 200 μM, the cell survival rate was less than 50% compared with that of the control group (Fig. 2a).

Next, we provided important evidence for nutritional zinc in bone tissue growth, especially along with osteoclast-mediated bone resorption. It has been previously demonstrated that Zn^2+^ exhibit dose-dependent toxicity in BMMs, with high concentrations leading to cell death. The effect of zinc on osteoclast precursor BMMs was evaluated in a simulated zinc microenvironment (where the final zinc concentrations in the culture medium were 0, 12.5, 25, 50, and 100 μM). After treatment with different concentrations of zinc, the mRNA expression levels of osteoclastogenesis-related genes, such as Trap, NFATC, and CTR, were detected. Specifically, low concentrations of nutritional zinc did not significantly inhibit osteoclast formation. With an increase in zinc concentration, the mRNA expression of osteoclast-related markers in the Zn^2+^– 25 and Zn^2+^– 50 groups decreased significantly compared with that in the control group (Fig. 2b).

We induced BMM differentiation into osteoclasts in medium containing different concentrations of Zn^2+^ by adding receptor activator of nuclear factor kappa-Β ligand (RANKL) and macrophage colony-stimulating factor (M-CSF) simultaneously; a lack of RANKL was considered a negative control for induction. Induction was halted when a continuous bullae-like structure was observed in the positive control group under an optical microscope. Tartrate-resistant acid phosphatase (TRAP) staining showed that osteoclast differentiation induced by M-CSF and RANKL was inhibited by zinc addition. Among them, 50 μM zinc exhibited the most obvious inhibition, with almost no multinucleated bullae cells formed in the visual field, and trap staining was negative (Fig. 2c). As the zinc concentration decreased, nuclei aggregated and osteoclasts were induced. This is consistent with the generation trend of the osteoclast actin ring observed under the confocal view (Fig. 2d, e).

Overactivation of osteoclasts can disrupt bone homeostasis and lead to bone resorption disorders, such as osteoporosis, in postmenopausal women or aged people. Here, we selected 50 μM as the optimal Zn^2+^ concentration to improve the osteogenic-osteoclast dialogue in the elderly population.

### Isolation and characterization of small extracellular vesicles from senile osteoporosis

**Fig. 3:**
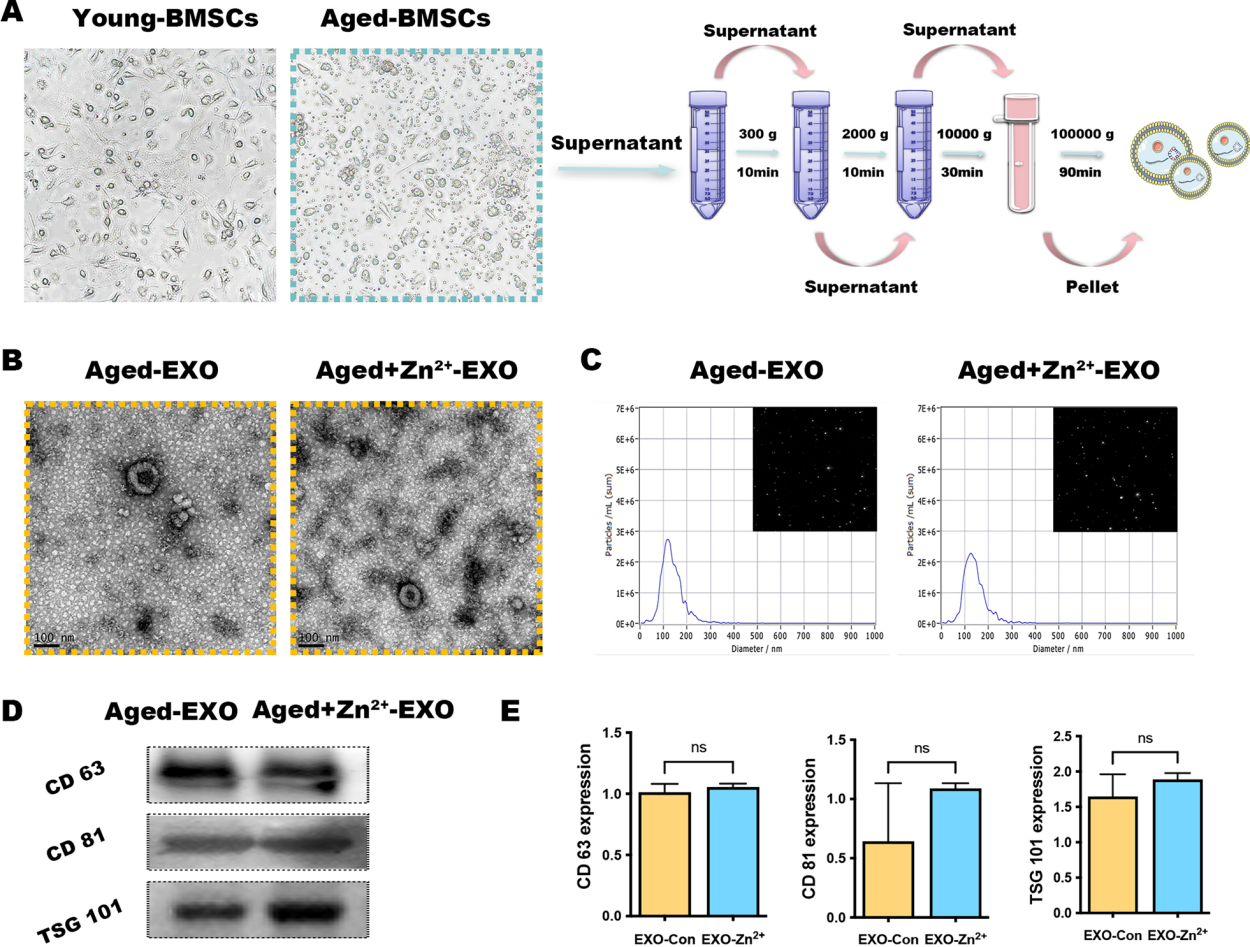
Identification of aged exosomes with and without zinc addition (AZ-EVs and A-EVs, respectively). **a** Representative images showing the spindle-like morphology of bone marrow mesenchymal stem cells and their extraction by ultracentrifugation. **b** Physical parameters of the purified exosomes under transmission electron microscopy and (**c**) nanoparticle tracking analysis. **d**, **e** Surface markers were scrutinized by western blotting

An estimated 17% of the world's population is at risk of insufficient zinc intake, with this estimate rising to 35–45% in older adults [25]. We performed primary cell culture of young and aged BMSCs, and morphological observations revealed a spindle-like shape. However, the growth rate of 20-month-old rat BMSCs significantly slowed, and colony formation was significantly reduced (Fig. 3a). Aged BMSCs were cultured to the P3 generation and treated with or without 50 μM zinc, and ultracentrifugation was used to separate exosomes from cells (those without zinc were named Aged-EXO or A-EVs for short; those with zinc were named Aged- Zn^2+^-EXO, abbreviated as AZ-EVs).

Thereafter, we used various methods to characterize the extracted EVs. Transmission electron microscopy (TEM) was used to characterize individual vesicles in each group, and spherical microvesicle structures were observed. The isolated exosomes exhibited homogeneity and oval morphology (Fig. 3b). Furthermore, examination of the average diameter of exosomes using nanoparticle tracking analysis (NTA) revealed that most of the populations were in the 90–130 nm range (Fig. 3c). In addition, western blot analysis showed that the exosome surface markers CD81, CD63, and TSG101 were expressed on EVs (Fig. 3d) also they were quantified (Fig. 3e). The EVs of senescent cells did not differ in shape and size from the EVs of ordinary cultured cells. Moreover, the addition of 50 μM zinc did not affect the biological properties of EVs. In general, the above results confirmed that EVs were successfully isolated from Aged-BMSCs.

To evaluate the effect of A-EVs and AZ-EVs secreted by aging rats on the osteogenic dialogue of BMSC-BMMs, we first examined whether green lipophilic dye-labeled A-EVs and AZ-EVs could be internalized by osteoclast precursors. The confocal microscopy images in Fig. 3a show that after 4 h of treatment with A-EVs and AZ-EVs, a large green fluorescence signal appeared in the perinuclear region of BMMs, indicating that BMMs successfully internalized these EVs. Nuclei were stained with blue fluorescence using 4′,6-diamidino-2-phenylindole (DAPI). The results showed that EVs secreted by senescent cells did not affect their endocytosis by other cells.

### AZ-EVs favor osteogenic-osteoclast imbalance in vitro

**Fig. 4:**
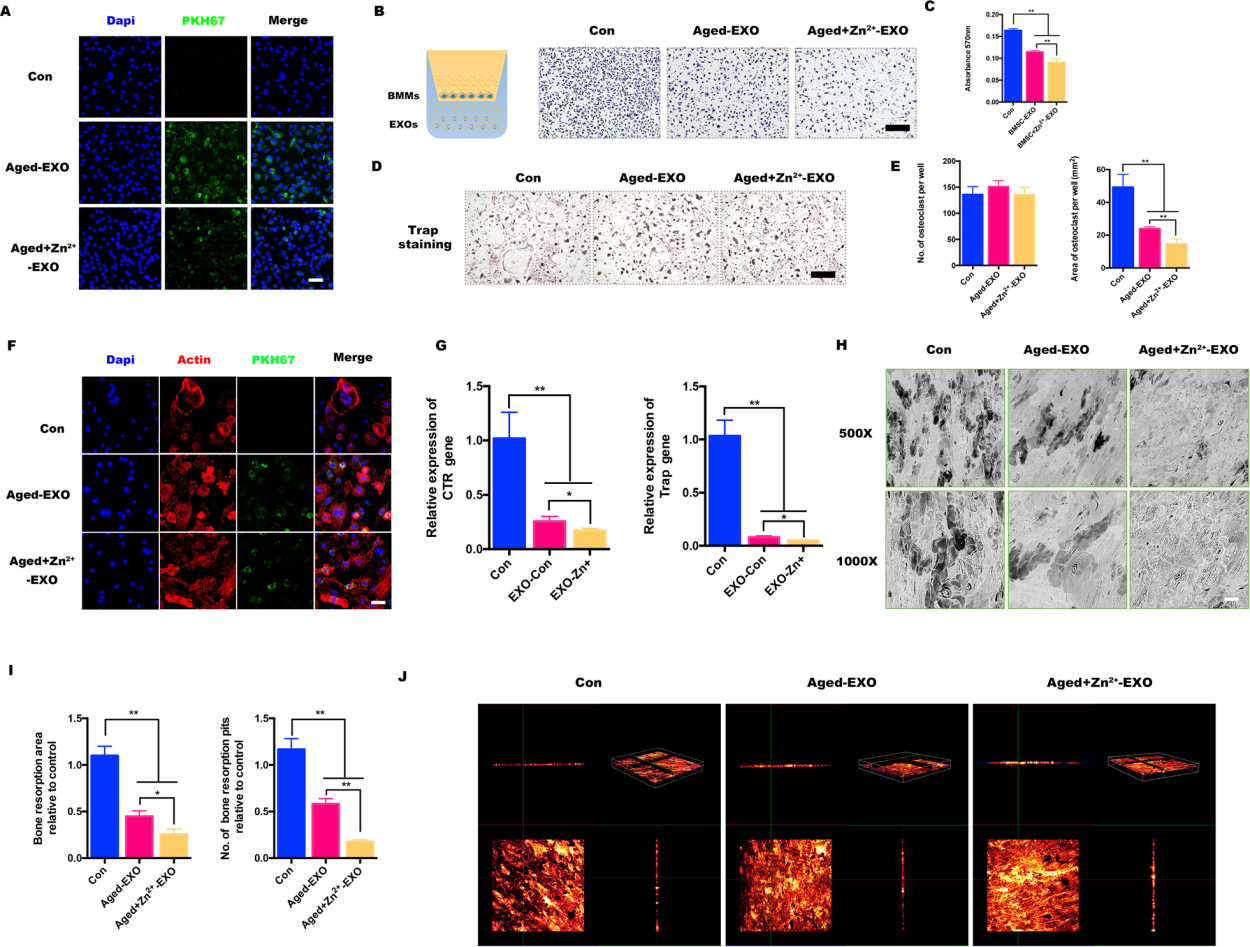
Aged-bone marrow mesenchymal stem cell (BMSC) exosomes target osteoclasts in vitro. **a** Colocalization of labelled BMSC-exosomes and bone marrow monocyte (BMM) nuclei. **b**, **c** Schematic illustration of the Transwell assay. The bottom membrane was stained with crystal violet after 24-h culture in vitro. **d**, **e** Representative images of TRAP-stained multinucleated osteoclasts treated with aged exosomes without zinc (A-EXOs) or aged exosomes with zinc addition (AZ-EXOs) and the area and number of TRAP‐positive cells. **f** Podosomal actin belts in osteoclasts treated with A-EXOs or AZ-EXOs; nuclei were counterstained with 4′,6-diamidino-2-phenylindole. **g** Semi-quantitative statistical analysis of osteoclastogenesis-related expression (TRAP and CTR). **h**, **i** Representative scanning electron microscopy images of bone resorption pits on different bovine bone slices and their resorption area. **j** Confocal microscopy observation of 3D reconstruction on different bovine bone slices (n = 3, *p < 0.05, **p < 0.01)

Transwell co-culture systems were established in which BMMs were seeded in the Transwell upper chamber and either A-EXOs, AZ-EXOs, or nothing (control) was added. The results showed that significantly more cells cultured with A-EXOs or without exosomes migrated to the underside of the porous membrane (Fig. 4b). In other words, AZ-EXOs inhibited the migration of osteoclast precursors, and osteoclasts received inhibitory signals from senescent BMSCs in a high-zinc environment, making their generation more difficult. The cells stained with crystal violet in the lower chamber were dissolved in absolute ethanol, the optical density value was determined, and the results were found to be statistically significant (Fig. 4c).

In addition, the ability of zinc to inhibit osteoclast differentiation of BMMs was explored. Osteoclast formation was detected by TRAP staining, and the number of large-area Trap-positive multinucleated cells formed was counted. Compared with that in the control group, both A-EXO and AZ-EXO inhibited the generation of OC (Fig. 4d); the generation of multi-core OC was almost absent in the AZ-EXO group, and the number was also greatly reduced (Fig. 4e).

A well-formed podal actin band around osteoclasts is the morphological hallmark of well-distributed osteoclasts. Therefore, we investigated whether A-EXO or AZ-EXO treatment affects the formation of cytoskeletal podosome actin ribbons. Confocal microscopy observations showed that only the control group exhibited a strong and clear podosomal actin belt, whereas the other two groups had almost none. The nuclei were stained with DAPI, and the results showed that the BMMs in the AZ-EXO-treated group had fusion defects (Fig. 4f). We investigated the expression profiles of specific marker genes required for osteoclastogenesis using qPCR analysis. The expression of the bone resorption genes CTR and Trap was significantly decreased in the AZ-EXO group, which was consistent with previous results (Fig. 4g).

The BMMs were seeded onto Osteo Assay Stripwell plates that simulated bone calcium phosphate coating, and the calcium phosphate bone chips were widely absorbed in the control group, with a large area of black shadow. Although random small resorption pits were observed in the A-EXO-treated group, almost no bone resorption was observed in the AZ-EXO-treated group (Fig. 4h). After the treatment, the absorption area was reduced by approximately 40% and 80%, respectively (Fig. 4i). The bone slices were stained with Alizarin Red S, and three-dimensional reconstruction was performed using a confocal microscope to observe the pits in the bone slices. In the control group, there was a large area of bone depression, and the side view showed that the bone fragment was discontinuous, whereas in the AZ-EXO group, the bone fragment was barely resorbed, and the side view showed that the bone fragment was continuous (Fig. 4j).

In conclusion, our study demonstrated that exosomes from aged-BMSCs in a high-zinc environment can suppress osteoclast differentiation and bone destruction in vitro, especially when compared with common aged BMSCs. Even in an aging environment, the addition of zinc can effectively regulate the osteogenesis-osteoclast dialogue via EVs and inhibit the excessive activation of osteoclasts, which provides a theoretical basis and guidance for combating osteoporosis caused by aging.

### Identification of differentially expressed miRNAs by bioinformatics in senile BMSC exosomes

It is well known that EVs can carry biologically active substances, such as miRNAs and proteins, and transport them to target cells secreted by host cells to further regulate the function and viability of target cells. Among them, miRNAs are the most well-studied and are highly conserved among species [26, 27]. miRNAs play an important role in regulating gene expression and biological function by binding to the 3’-UTR or amino acid-coding sequences of target genes [28]. To explore the potential mechanism by which the trace element zinc inhibits osteoclast differentiation in a high-zinc environment by regulating the release of miRNA from aged BMSC-EVs, we further identified differentially expressed miRNAs between A-EXOs and AZ-EXOs through miRNA sequencing studies.

**Fig. 5:**
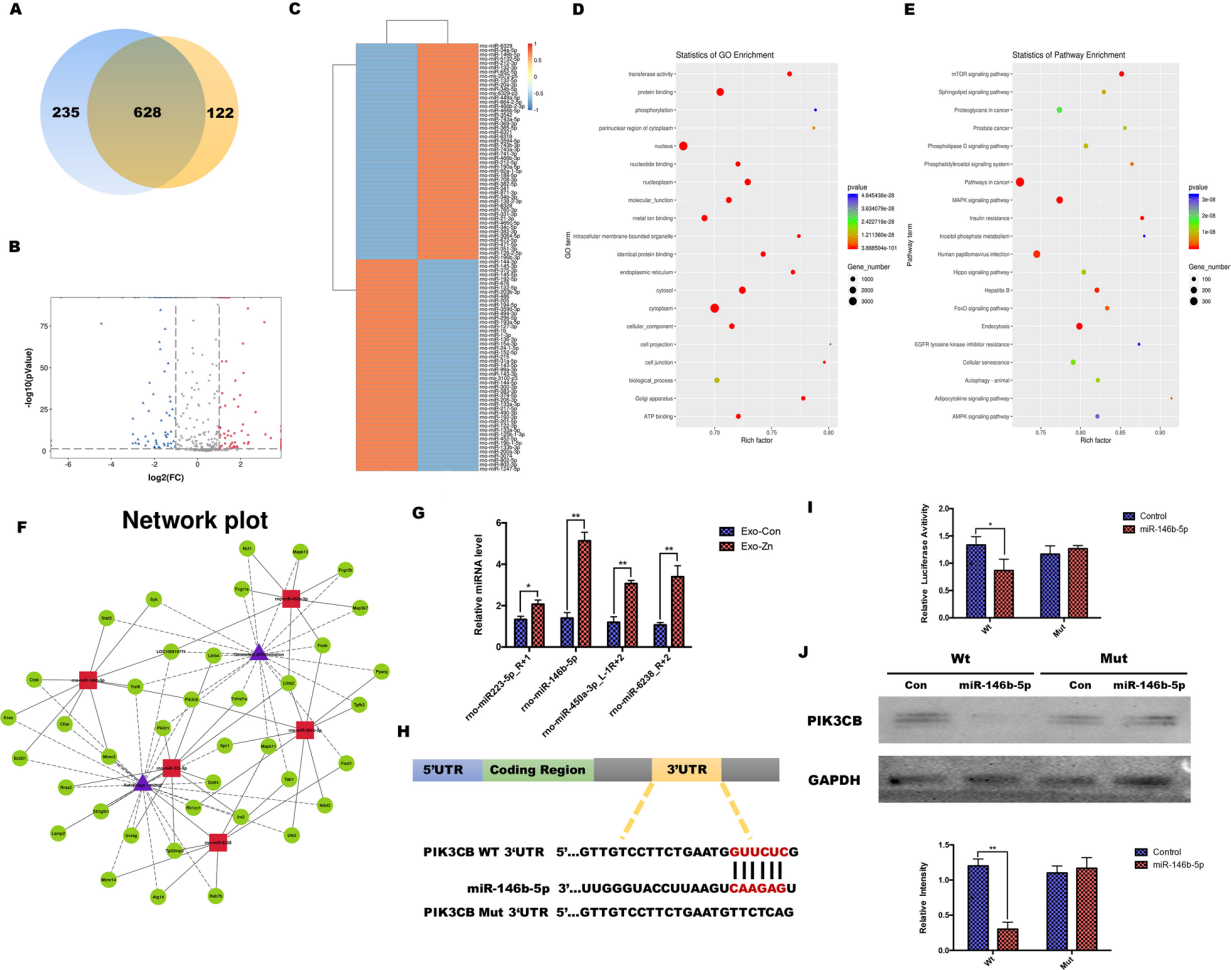
MicroRNAs (miRNAs) identified in aged extracellular vesicles. **a**, **b** Venn diagram and volcano plot analysis of the total miRNAs from aged exosomes with zinc (AZ-EXOs) and without zinc (A-EXOs). **c** Heatmap of differentially expressed miRNAs between AZ-EXOs and A-EXOs. **d**, **e** Bubble plot showing Gene Ontology enrichment in molecular function and signaling pathway enrichment in Kyoto Encyclopedia of Genes and Genomes pathways. **f** Network plot of the interactions of enriched biological pathways. **g** High expression of miRNA-450a-3p, miRNA-301b-3p, miRNA-223-5p, and miRNA-6328 in AZ-EXOs was confirmed using RT-PCR. **h**, **i** Dual-luciferase assay was conducted for wild-type and mutated miR-146b-5p recognition sites within the PI3CK 3’UTR. **j** Western blot and quantitative analysis of PI3CK protein levels (n = 3, *p < 0.05, **p < 0.01)

As shown in Fig. 5a, b, Venn diagram and volcano plot analysis showed that there were significant differences in miRNA expression between A-EXOs and AZ-EXOs. Among these, 628 miRNAs were co-expressed in the two groups, 235 were expressed by A-EXOs alone, and 122 were expressed only in AZ-EXOs. Our screening criteria were |log2(FC)|> 2.5 (FC: fold change) and *P* < 0.001, and cluster heatmap analysis revealed significantly differentially expressed miRNAs in the A-EXO and AZ-EXO groups (Fig. 5c). We then assessed significantly enriched Kyoto Encyclopedia of Genes and Genomes (KEGG) pathways and Gene Ontology (GO) terms. The differentially expressed miRNAs were mainly related to the GO functional annotations of biological processes, including metal ion and protein binding (Fig. 5d). This indicates that in the high zinc ion microenvironment, metal ions can enter stem cells and thereby regulate the secretion of EXOs from stem cells, activating mental binding and causing downstream reactions. All significantly affected KEGG pathways were identified by comparing the signaling pathways involved in osteoclast differentiation. Among them, the mTor, mitogen-activated protein kinase, and AMP-activated protein kinase signaling pathways are reported to be closely related to bone repair and regeneration as well as osteoclast differentiation (Fig. 5e).

Of all the biological processes identified, we were particularly interested in the enrichment of osteoclast differentiation with the activation of autophagy, as it has been reported to regulate the osteogenic-osteoclast balance [29, 30]. Using biological analyses, we mapped a rich network of biological pathway interactions. Five highly correlated miRNAs, including miRNA-146b-5p, miRNA-450a-3p, miRNA-301b-3p, miRNA-223-5p, and miRNA-6328, were screened (Fig. 5f). To further explore the differences in miRNA expression in osteoclast-related pathways and autophagy-related biological processes, we transfected A-EXOs and AZ-EXOs into the BMMs. The expression levels of miRNAs in cells were detected by real-time PCR, and it was found that the expression of miRNA-146b-5p had the most significant difference, which was also consistent with the results of the bioinformatics analysis (Fig. 5g). These results indicated that miR-146b-5p expression was elevated in BMSCs, and that BMSC-derived exosomal miR-144-5p could be internalized by BMMs. To further investigate the potential mechanism of miR-146b-5p secretion by AZ-EXOs in osteoclastogenesis, differentiation, and autophagy, we predicted potential target genes of miR-146b-5p using the TargetScan and miRanda databases. Among the predicted target genes, PI3CK was strongly correlated with osteoclast autophagic activity. Bioinformatics analysis revealed that miR-146b-5p could bind to PI3CK 3'UTR (Fig. 5h). To verify that the PI3CK 3'UTR is a direct target of miR-146b-5p, we analyzed the transfected BMMs using a luciferase reporter assay. The PI3CK 3'UTR containing the predicted and mutated PI3CK 3'UTRs were cloned and transfected into BMM cells. Luciferase activity was reduced when transfected cells were incubated with miR-146b-5p, and the response was abolished by mutating the target sites in the 3ʹUTR of PI3CK. When downregulated miR-146b-5p was co-transfected with the BMM wild-type luciferase construct, the relative luciferase activity decreased (Fig. 5i). Western blot analysis also showed the protein level of PI3CK was significantly inhibited (Fig. 5j). Together, these results suggest that miRNA-146b-5p secreted by AZ-EXOs plays a crucial role in regulating osteoclast differentiation.

### miR‐146b‐5p regulates osteogenesis differentiation by targeting the PTEN/AKT/mTor pathway

**Fig. 6:**
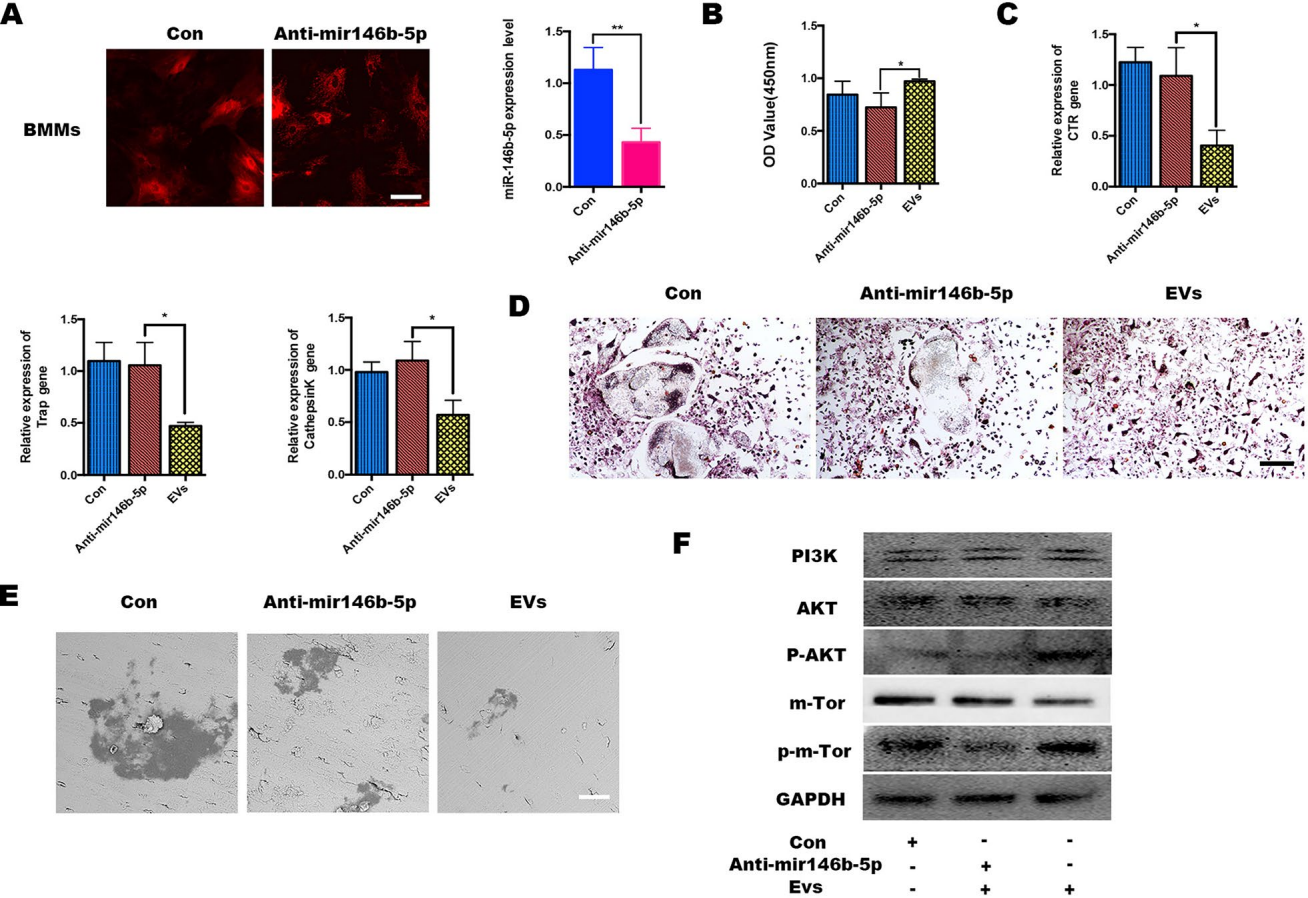
miR-146b-5p silencing attenuated the effect of aged exosomes with zinc (AZ-EXOs) in bone marrow monocytes (BMMs). **a** BMMs were treated with miRNA-146b-5p-inhibitor for 6 h and the expression was tested by RT-PCR. **b** Cell counting kit-8 detection of BMMs with or without inhibitors. **c** RNA expression levels of CTR/TRAP/CTSK in BMMs. **d** TRAP staining of osteoclasts. **e** Representative scanning electron microscopy images of the bone resorption pits. **d** Western blot analysis of the phosphatase and tensin homolog (PTEN)/protein kinase B (AKT)/mammalian target of rapamycin (mTOR) pathway and its phosphorylation activation.

To investigate the effect of miRNA-146b-5p in AZ-EXOs on the biological behavior of BMMs, we transfected BMMs with miRNA-146b-5p-inhibitor and inhibitor-NC (labeled Con), both labeled red fluorescence. RT-PCR results showed that the level of miRNA-146b-5p in BMMs decreased significantly after transfection with the miRNA-146b-5p-inhibitor (Fig. 6a). The CCK-8 assay showed that the proliferation ability of BMMs transfected with miRNA-146b-5p was slightly lower than that in the AZ-EXO group transfected alone (Fig. 6b). Next, we sought to assess whether miRNA-146b-5p-inhibitor might influence AZ-EXOs to mediate the differentiation of BMMs. BMMs were co-transfected with miRNA-146b-5p-inhibitor and inhibitor-NC, and RT-PCR results showed that compared with those in the miRNA-146b-5p-inhibitor group, osteoclast differentiation-related genes such as Trap, CTSK, and CTR were significantly reduced (Fig. 6c). The formation of multinucleated giant cells in the AZ-EXO group was also significantly inhibited, while differentiated osteoclasts were observed after reducing the level of miRNA-146b-5p in BMMs in the Trap staining (Fig. 6d). The above results demonstrate that high expression of miRNA-146b-5p in AZ-EXOs inhibits the differentiation of BMMs into osteoclasts. The bone resorption experiments also showed that the inhibited level of miRNA-146b-5p activates BMMs into osteoclast differentiation (Fig. 6e). Finally, western blot analysis indicated that phosphorylation activation of the AKT and mTOR signaling pathways was significantly inhibited by the addition of miRNA-146b-5p-inhibitor, indicating that miRNA-146b-5p activates the mTOR pathway through the miRNA-146b-5p-AKT signaling axis and further inhibits the differentiation of BMMs into osteoclasts (Fig. 6f). Studies have shown that miR-146b can target the phosphatase and tensin homolog (PTEN)/AKT/mTOR signaling pathway, which is consistent with the results of our previous biological analysis [31]. In conclusion, during bone resorption, AZ-EVs secreted by aging BMSCs are released from the bone matrix into the bone marrow, and miRNAs delivered by AZ-EVs are taken up by osteoclast precursors, mediating over-activation of osteoclasts and resulting in an imbalance of bone metabolism and enhancement of bone regeneration in osteoporosis.

### Constructing a smart metal implant to regulate in vivo integration in senile osteoporosis

**Fig. 7:**
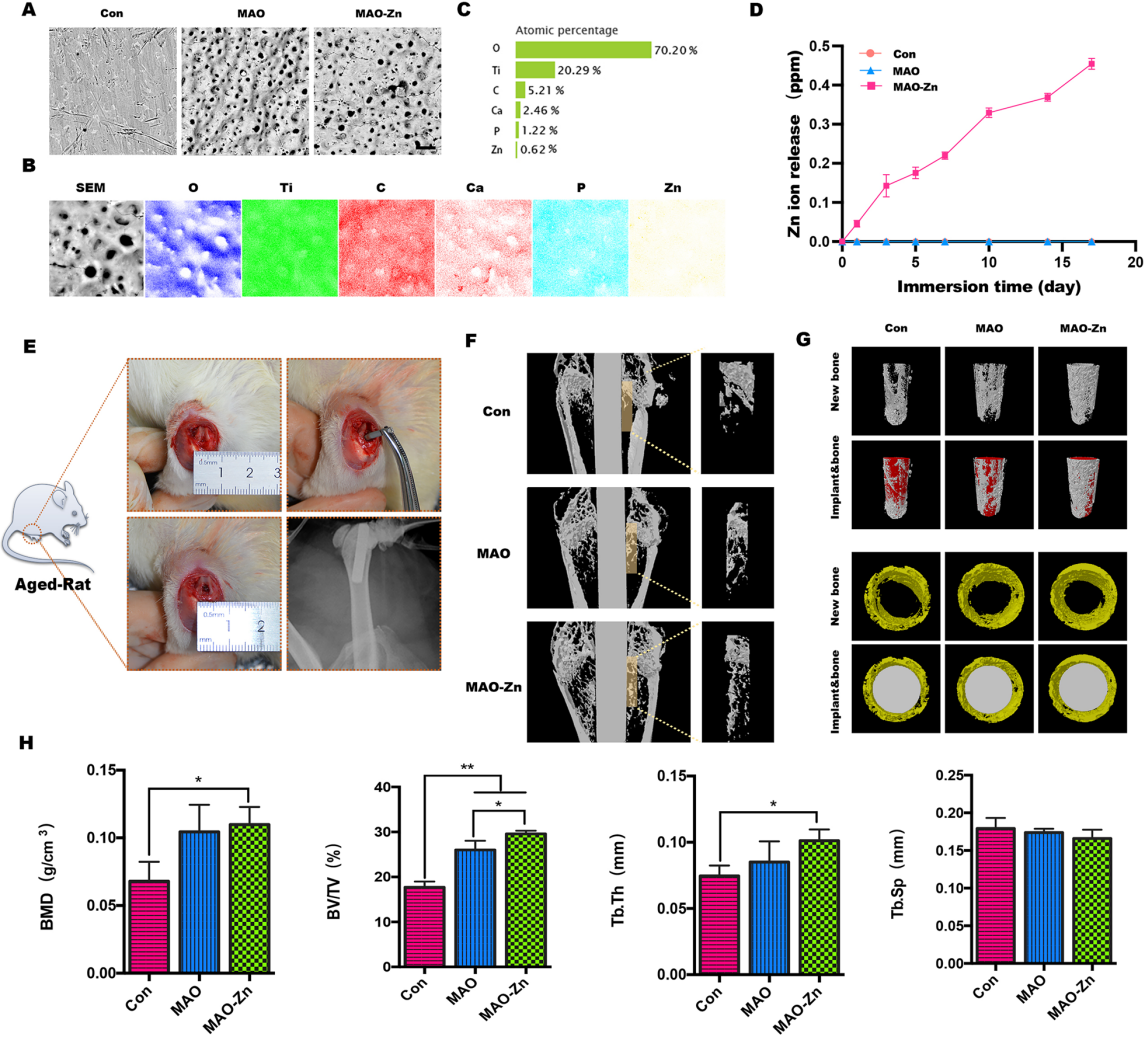
Analysis of the products after knee implantation in aged rats. **a** Scanning electron microscopy images of the implant surfaces. **b**, **c** Energy-dispersive X-ray spectroscopy analysis of the micro-arc oxidation implant with zinc acetate addition to the electrolyte (MAO-Zn^2+^) group. **d** Cumulative concentrations of Zn.^2+^ released from MAO-Zn during the immersion in a PBS solution at 37 °C. **e** Model diagram and X-ray image of the metal implant model of the aging rat femur. **f**, **g** Promoting osteointegration in vivo detected by microCT in 2D or 3D views. **h** Statistical analysis of bone mineral density (BMD), bone volume to tissue volume (BV/TV), trabecular thickness (Tb.Th), and trabecular separation (Tb.sp) using the acquired microCT data for each group (n = 3, *p < 0.05, **p < 0.01)

Inspired by the above results, we attempted to fix a zinc coating on the surface of micro-implants using micro-arc oxidation technology and studied its effect on the surrounding osseointegration and vital activities of osteoclasts in senile osteoporosis. The micro-implants used in the experiment were 1 cm in length and 1 mm in diameter. Naturally aged rats were selected, and implants were implanted at the knee joint (Fig. 7e). Unmodified pure titanium implants (Con) and micro-arc oxidation implants (MAO) without zinc in the electrolyte were used as controls, and zinc acetate was added to the electrolyte in the experimental group (MAO-Zn) per the protocol established in our previous study [32].

First, changes in the surface roughness of the implants after zinc modification were observed using a scanning electron microscope (Fig. 7a). The results show that the surface roughness increased in the MAO and MAO-Zn groups. Elemental mapping by energy-dispersive X-ray spectroscopy (EDS) revealed that zinc was homogeneously modified on the implant surface (Fig. 7b). The composition of the surface elements was determined using X-ray photoelectron spectroscopy. The quantitative analysis results showed that the mass percentage of zinc on the surface of MAO-Zn was 0.62%, indicating that it was successfully modified on the surface of the implant (Fig. 7c). The amounts of Zn released from Con, MAO and MAO-Zn coatings were measured, as shown in Fig. 7d. With immersion prolonged, cumulated Zn from MAO-Zn increase while there’s no Zn release in both Con and MAO group.

The 2D image around the implant was reconstructed using a software program, a cylindrical area with a diameter of 1.5 mm was selected for 3D reconstruction around the implant, and the newly formed bone tissue was quantitatively analyzed. The results were consistent with the in vitro observations that although the naturally aged rats showed obvious trabecular osteoporosis on imaging, in the MAO-Zn group, the newly formed bone tissue was still firmly attached to the peri-implant, and the area of bone neogenesis was the widest among the groups. The CON group had relatively little new bone tissue, which also indicated that osseointegration in aging osteoporosis was difficult (Fig. 7f). To accurately observe the new bone around the whole implant, trabecular bone formation around the implant was observed from different angles after 3D reconstruction, which was consistent with the above results (Fig. 7g). In addition, bone mineral density, bone volume to tissue volume, trabecular separation, and trabecular thickness were quantified in the CON, MAO, and MAO- Zn groups, and it was found that the MAO- Zn group exhibited the best trabecular structural features of the new bone (Fig. 7e).

**Fig. 8:**
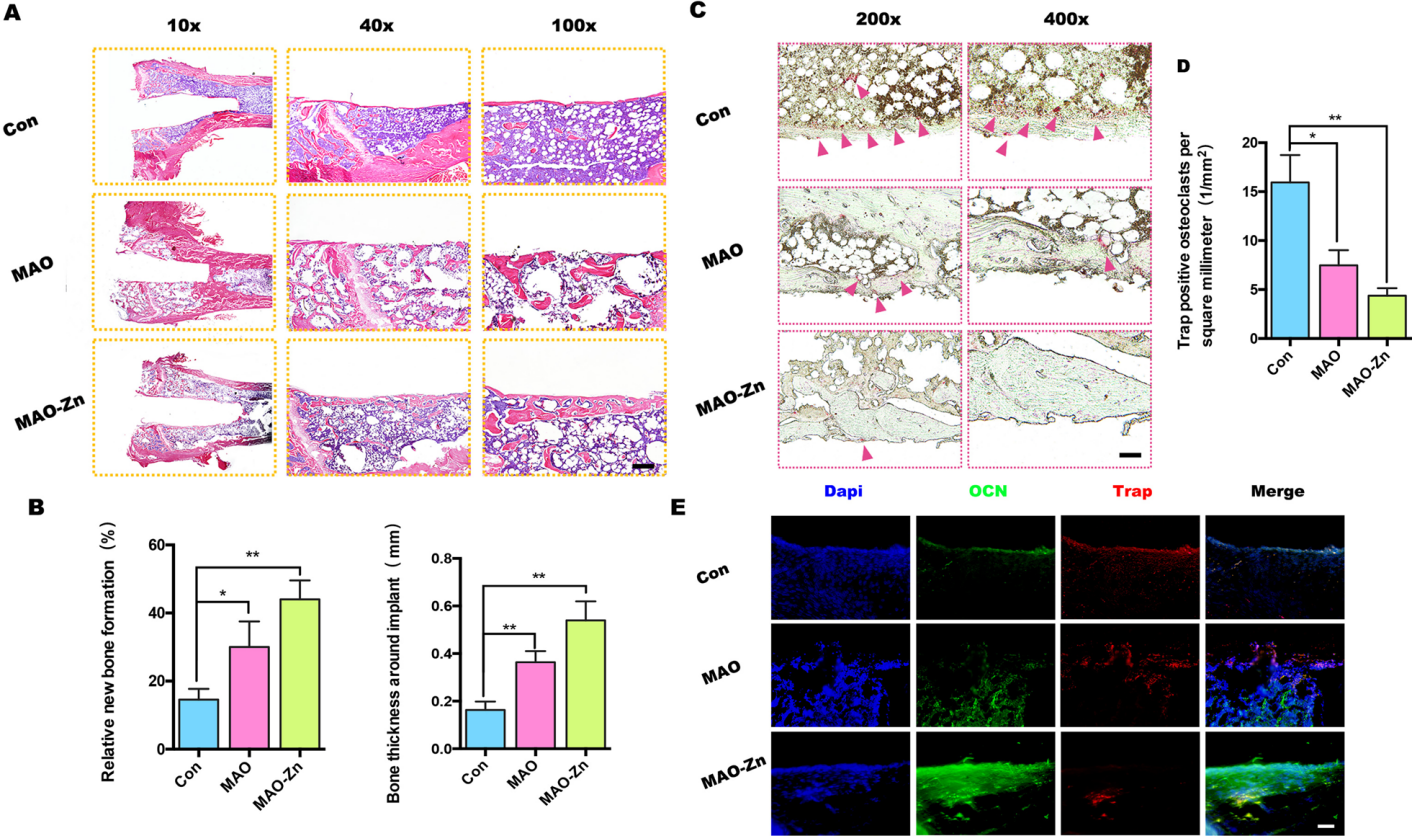
Histological evaluation of senile osteoporosis osseointegration. **a**, **b** Representative histological sections of hematoxylin and eosin staining after 8 weeks and quantitative analysis. **c**, **d** Representative histological sections of TRAP staining in vivo and quantitative analyses. **e** Immunohistochemical staining of osteocalcin (OCN) and Trap proteins in the right distal femur of both groups (n = 3, *p < 0.05, **p < 0.01)

Further, hematoxylin and eosin staining was used to detect new growth of the surrounding tissue of the implant. The results showed that although there was more vacuolar bone marrow matrix around the implant in the senile osteoporotic rats, new bone formation still occurred around the implant. In the MAO-Zn group, the new bone was almost continuous and the tissue was more complete (Fig. 8a); the thickness was the thickest, the degree of osseointegration was the highest, and the number of new bone trabeculae was the largest in this group (Fig. 8b). To verify whether this change is strongly correlated with the activity of osteoclasts, TRAP staining was used to detect the phenotype of osteoclasts around the implants. The osteoclasts around the implants were stained red, and histological quantitative analysis detected the lowest number of osteoclasts in the MAO-Zn^2+^ group (Fig. 8c, d). Colocalization analysis of the expression of the osteogenic marker osteocalcin (OCN) with TRAP immunohistochemistry revealed the most OCN-positive cells in the MAO-Zn-implanted femurs and the Trap-positive cells in this group were hardly distributed around the implant (Fig. 8e).

Taken together, the MAO- Zn coating synthesized by micro-arc oxidation acts as a reservoir for slow-release of zinc ions to maintain the long-term local high-concentration AZ-EXOs required for aging BMSCs to communicate with OC cells. Moreover, the direct inhibitory effect of zinc on OC was activated, which fully inhibited the generation and activity of osteoclasts, thereby promoting bone regeneration of titanium implants.

## Conclusion

In conclusion, the implant designed in this study directly inhibits the differentiation of BMMs towards osteoclasts by creating a high zinc atmosphere in aging bone and modulates the intercellular dialogue between senescent BMSC-BMMs by continuously changing the exosomal cargo (e.g., miRNAs) of aging BMSCs. This study provides a novel exosome-targeted orthopedic implant that can guide exosome cargo transport, thereby broadening the effective application of metal implants in the treatment of elderly osteoporotic populations.

## Methods

### Cell culture and animals: animals

All animals used in the experiments were provided by the Ninth People's Hospital Animal Center (Shanghai, China), and the experiments were conducted in accordance with the requirements of the Animal Welfare Law. All experimental procedures were approved by the Animal Care and Experiment Committee of Ninth People's Hospital. Male Sprague–Dawley (SD) rats were divided into two age groups: young (4 weeks) and old (20 months), and the specific culture method of BMSCs is shown outlined below.

### Cell culture and animals: cell culture

BMSCs were isolated from 4-week-old and 20-month-old SD rats. Old or young BMSCs were cultured as previously described in high-glucose Dulbecco’s modified Eagle’s medium (DMEM) (HyClone Laboratories, Inc, Logan, UT, USA) containing 10% fetal bovine serum (FBS), 100 U mL^−1^ penicillin, and 100 U mL^−1^ streptomycin (Gibco, Thermo Fisher Scientific, Waltham, MA, USA).

BMMs were prepared for osteoclast culture as previously described [33]. Briefly, primary BMMs were isolated immediately from the femurs and tibias of C57BL/6 mice. After 24 h, non-adherent monocytes were seeded in 96-well plates at 1 × 10^4^ cells/well in a medium containing 100 U mL^−1^ penicillin/streptomycin, 10% FBS, and 30 ng mL^−1^ M-CSF (PeproTech, Rocky Hill, NJ, USA) in α-minimum essential medium (Sigma-Aldrich, St. Louis, MO, USA). RANKL (100 ng ml^−1^; R&D Systems, Minneapolis, MN, USA) was added to the medium on day 3, and the medium was changed every day until multinucleated osteoclasts were observed (approximately 7 days). All cells were cultured in a humidified environment at 37 °C and 5% CO_2_.

### In vitro osteoclastogenesis assay: trap staining

All BMMs were cultured in 96-well plates, as previously described for the osteoclast culture method. We first determined the toxicity of Zn^2+^ (ZnCl_2_) using a CCK-8 assay (Dojindo Laboratories Inc., Kumamoto, Japan). After incubation for 2 h, the absorbance was detected at 450 nm, and cell viability was calculated. According to the results of the CCK-8 assay, different concentrations of zinc ions (0, 12.5, 25, 50, and 100 μM) were used to stimulate differentiation. The medium was changed daily until osteoclasts were induced in the positive control wells. The 96-well plate was gently rinsed with phosphate-buffered saline (PBS), fixed with 4% paraformaldehyde for 30 min, and stained with TRAP staining solution (Sigma-Aldrich; Merck KGaA) for 30 min at 37 °C. TRAP-positive cells with three or more nuclei were counted as multinucleated osteoclasts. Images were obtained using an optical microscope (Nikon, Tokyo, Japan). TRAP activity was quantified by densitometric analysis using ImageJ software (National Institutes of Health, Bethesda, MD, USA).

### In vitro osteoclastogenesis assay: actin ring assay

Immunofluorescence analysis of the podosomal actin belt was performed and osteoclastogenesis was induced using the same method. When osteoclasts appeared in the control wells, they were gently rinsed with PBS, permeabilized with 0.1% Triton X-100 (Sigma-Aldrich) for 30 min, and finally stained with rhodamine-conjugated phalloidin to detect cytoskeletal actin structure formation. The final images were obtained using a confocal laser microscope (Leica Camera AG, Wetzlar, Germany).

### In vitro* osteoclastogenesis assay*: real-time PCR detection

The relative gene expression levels during osteoclast differentiation were determined by quantitative real-time PCR. Total RNA from BMM-derived osteoclasts was extracted using TRIzol, and 2 μg of the extracted RNA was reverse transcribed into complementary DNA (cDNA) using a PrimeScript™ RT Kit (TaKaRa Bio, Shiga, Japan). The resulting cDNA was used as a template in a TB Green^®^ Premix Ex TaqTM Kit (TaKaRa Bio) for quantitative PCR using a quantitative real-time PCR system (LightCycler^®^ 480II; Roche, Basel, Switzerland). Where glyceraldehyde 3-phosphate dehydrogenase (GAPDH) was used as a reference gene, the relative gene expression levels were investigated using the 2^−ΔΔCt^ method. All assays were performed in triplicate. The mouse primer sequences used were as follows: GAPDH (5ʹ-GGTGAAGGTCGGTGTGA ACG-3ʹ, 5ʹ-CTCGCTCCTGGAAGATGGTG-3ʹ), Trap (5′-CAAAGAGATCGCCAGAACCG-3′ and 5′-GAGACGTTGCCAAGGTGATC-3), Nfatc1 (5ʹ-CCGTTGCTTCCAGAAAATAACA-3ʹ), 5ʹ‐TGTGGGATGTGAACTCG GAA‐3ʹ), CTR (5′‐TGCAGACAACTCTTGGTTTGG‐3′ and reverse, 5′‐TCGGTTTCTTCTCCTCTGGA‐3′).

### Extraction and identification of exosomes: extraction of exosomes.

Second-generation aged BMSCs were selected for exosome extraction with or without a zinc ion medium. When the BMSCs reached 60% confluence, they were replaced with a medium filled with 10% exosome-free serum (EXO-FBS-50A-1; System Biosciences, LLC, Palo Alto, CA, USA). Culture supernatants were collected after 48 h. The cell culture supernatant was centrifuged using ultracentrifugation technology as follows: 300×*g* for 10 min, 2000×*g* for 10 min, 10,000×*g* for 30 min, and ultracentrifugation at 100,000×*g* for 90 min (Beckman Coulter Inc., Brea, CA, USA). The exosome pellet was carefully resuspended in 100 μL PBS. Concentration quantification of exosomes was performed using a bicinchoninic acid (BCA) protein quantitation kit (23225; Thermo Fisher Scientific).

### Extraction and identification of exosomes: identification of exosomes

Nanoparticle tracking analysis (NTA; NanoSight Ltd., Salisbury, UK) was used to check the size of all exosome samples before their use, and transmission electron microscopy (TEM) was used to verify their morphology. To verify the isolation of exosomes from the serum, western blot analysis was performed using TSG101, CD9, and CD81, which are enriched in exosomes. Briefly, exosomes were lysed with 1× RIPA buffer containing a protease inhibitor cocktail (Roche) and quantified using a BCA Protein Assay Kit (Beyotime Institute of Biotechnology, Jiangsu, China). Sodium dodecyl-sulfate polyacrylamide gel electrophoresis (Gibco) was used to fractionate exosomes after they were denatured by boiling in cell buffer and then transferred to polyvinylidene difluoride membranes. Primary and secondary antibodies were added to the membranes overnight, followed by blocking with non-fat dry milk. The proteins were exposed to the film, developed, and fixed with an enhanced chemiluminescence reagent.

### Extraction and identification of exosomes: cellular uptake of exosomes

To detect the uptake of BMSC-EVs by BMMs, purified exosomes were labeled with a PKH67 green fluorescent labeling kit (Sigma-Aldrich), as recommended by the manufacturer. Labeled BMSC-EVs were added to BMMs and co-cultured for 8 h. BMMs were fixed in 4% paraformaldehyde, stained with DAPI, and observed by laser confocal microscopy.

### Aged BMSC-exosomes and osteoclastic differentiation: influence of exosomes on BMM migration

The concentration of exosomes was adjusted to a final concentration of 50 μg mL^−1^, and the effect of EVs on the migration of osteoclast precursor BMMs was detected using a Transwell co-culture model. First, 200 μL of DMEM-cultured BMMs were added to the upper chamber of the Transwell, and conditioned medium containing exosomes was added to the lower chamber. After 12 h, cells in the upper chamber were wiped with a sterile cotton swab. Cells on the lower surface of the Transwell were fixed and stained with crystal violet, and the cells were observed and photographed under a light microscope (Nikon). Crystal violet was dissolved in absolute ethanol, and the absorbance was read at 570 nm.

### Aged BMSC-exosomes and osteoclastic differentiation: influence of exosomes on osteoclast induction

The effect of EVs on the induction of osteoclasts was detected using TRAP staining and an actin ring assay, as previously described. The concentration of the exosomes was 50 μg mL^−1^. Real-time PCR detection was performed to determine the effect of BMSC-exosomes on osteoclast induction.

### Aged BMSC-exosomes and osteoclastic differentiation: bone resorption assay

To detect the effect of BMSC-exosomes on the resorptive function of osteoclasts in vitro, BMMs were seeded on bone‐mimicking calcium phosphate‐coated osteo assay Stripwell plates (Corning, Corning, NY, USA). During culture, fresh medium containing M-CSF, RANKL, and exosomes was supplemented once per day. After 10 days, the surface cells on the bone slices were removed by ultrasound, the absorption pits were imaged by using SEM(), and the pit area was quantified using the ImageJ software. Bone-mimicking calcium phosphate-coated osteo assay Stripwell plates were stained with Alizarin Red staining solution and photographed using a confocal laser microscope (Leica Camera AG).

### Differential expression analysis of miRNAs: ExoRNA library construction and sequencing

Small RNA sequencing was performed using a commercial service (RiboBio, Guangdong, China). Briefly, total RNA was extracted from BMSC-exosomes or BMSC-Zn^2+^-exosomes and purified using an RNeasy mini kit (Qiagen, Hilden, Germany). cDNA was synthesized by reverse transcription and amplified by PCR, miRNA libraries were prepared and sequenced, and raw data were collected using the Illumina NextSeq 500 platform (Illumina, San Diego, CA, USA). Differentially expressed miRNAs were identified under the criteria of fold change > 1.0 or < 1.0, and *p*-value < 0.05. Heatmaps and volcano plots were drawn according to the differential miRNAs.

### Differential expression analysis of miRNAs: miRNA analysis and target gene prediction

GO and KEGG pathway term enrichment analysis were analyzed using DAVID Bioinformatics (Laboratory of Immunopathogenesis and Bioinformatics, Frederick, MD, USA). The target genes of miR-146b-5p were predicted using the miRDB, TargetScan, starBase, and miRPathDB databases. KEGG analysis, GO analysis, and PPI network mapping were performed on the target genes using the DAVID and STRING databases.

### Differential expression analysis of miRNAs: luciferase reporter assay and transfection

293T cells were seeded into 96-well plates and cultured in an incubator. Luciferase reporter vectors, including pmir-report-PIK3CB wt and pmir-report-PIK3CB mut, were transfected into HEK-293T cells. After 48 h of culture, the original medium was aspirated, the cells were washed with PBS, and the PBS was discarded. Next, 500 μL of 1 × passive lysis buffer was added to each well and shaken gently at room temperature for 15 min. The lysate was transferred to a 96-well assay plate, 20 μL lysate was added to each well, 100 μL luciferase assay reagent II was added to detect firefly luciferase, and 100 μL stop reagent was added to detect Renilla luciferase activity. The relative fluorescence values of the samples were calculated as firefly luciferase/Renilla luciferase.

### Silencing osteoclast activity using miR-146b-5p inhibitor

Lyophilized miR-146b-5p inhibitor and miR-146b-5p NC were prepared in a 20 μM working solution with RNase-free water. Cells were plated, and BMMs were cultured in serum-free medium and treated with 100 nM miR-146b-5p inhibitor or miR-146b-5p NCs for 6 h in a hot incubator under 5% CO_2_. Cellular miRNA was extracted and the transfection efficiency was detected by qRT-PCR. BMM cell proliferation was detected using the CCK-8 method, osteoclast differentiation was detected by qRT-PCR and TRAP staining, and its related downstream pathways were detected by western blotting.

### In vivo animal experiment: specimen preparation and characteristics

During the in vivo experiments, 10 × 10 × 0.1 mm pure titanium plates (99.6% purity) or 10 mm diameter, 1 cm long pure titanium rods (99.6% purity) were used and polished with 320, 800, and 1200 grit SiC sandpaper. The micro-arc oxidation (MAO) method was used for the processing. Briefly, the titanium plate/rod was placed in the MAO reactor, and the electrolyte contained calcium acetate monohydrate (C_4_H_6_CaO_4_H_2_O; 0.1 M), sodium dihydrogen phosphate dihydrate (NaH_2_PO_4_ 2H_2_O; 0.03 M) and CH_3_COO 2 Zn. The final concentration of Zn^2+^ in the electrolyte was 50 μM *6 (for the reasons for this specific configuration, please refer to our previous work). The electrical parameters were 400 V and 50 Hz, with a reaction time of 10 min. SEM images were obtained using a scanning electron microscope (Phenom, China), and the elements on the surface were detected using EDS accessories. The ionic release kinetics of Zn from MAO-Zn coatings were investigated. In short, each sample was immersed in 10ml phosphate buffered saline (PBS) at 37 °C. The total PBS was collected using a pipette and replaced with fresh PBS at intervals of 1, 3, 5, 7, 14 and 17 day. The released dosages of Zn were analysed using inductively coupled plasma mass spectroscopy (ICP-MS, PerkinElmer, ELAN DRC-e).

### In vivo animal experiment: establishment of a rat femoral bone defect model

Twenty-four aged rats (20 months) were randomly divided into three groups: pure titanium, MAO, and MAO with zinc addition (MAO-Zn). After anesthesia with isoflurane, the surgical area was shaved and disinfected with povidone-iodine and alcohol. A small incision was made along the side of the femoral knee joint and blunt dissection was performed to expose the femoral side of the knee joint. The implant cavity (Φ1 mm) was prepared by flushing with precooled sterile saline using the pilot drill of a dental handpiece. The implants were then tapped into the drilled hole and the soft tissue was sutured. Finally, an intramuscular injection of penicillin (100 000 IU) was administered daily for 3 days after surgery to prevent infection. Eight weeks after surgery, the aged rats were euthanized with an intraperitoneal overdose of pentobarbital.

### In vivo animal experiment: bone micro-CT evaluation

A micro-CT system (μCT50; Scanco Medical, Brüttisellen, Switzerland) was used to quantify gross bone morphology and microarchitecture. The scanning interval was 15 µm per layer. The implant and its surrounding femur were scanned, and the area of interest was delineated as a 200 µm circular area around the implant. Bone mineral density, bone tissue volume/total tissue volume, trabecular thickness, and trabecular separation were assessed using image analysis software.

### In vivo animal experiment: histological evaluation

The implants were pushed out from the femoral knee joints of aged rats using a push–pull machine, and the remaining bone tissue samples were fixed with formalin for 48 h. It takes approximately one month for ethylenediaminetetraacetic acid to decalcify so that the needle can be easily inserted into the bone tissue. The samples were dehydrated in graded alcohol and embedded in paraffin, and thin slices with a thickness of 5 μm were prepared along the long axis of the implant using a paraffin microtome (Leica Camera AG).

Peri-implant bone histology was detected using hematoxylin and eosin, TRAP, and immunofluorescence chemical colocalization staining. The specific method has been detailed previously, and for immunocolocalization, the primary antibodies used were OCN (# MAB1419; Bio-Techne Corporation, Minneapolis, MN, USA) and Trap (ab191406; Abcam, Cambridge, UK).

### Statistics and reproducibility

All data are presented as mean ± standard deviation. Statistical analysis was performed by one-way analysis of variance or a *T*-test using the GraphPad Prism 8.0 software (GraphPad Software, San Diego, CA, USA). Statistical significance was set at *p* < 0.05. All data and image analyses were performed independently at least three times, with similar results.

## Data Availability

The authors confirm that the data supporting the findings of this study are available within the article [and/or] its supplementary materials.
